# Arteriovenous Hemangiomas: A Manifestation of Chronic Liver Disease

**DOI:** 10.7759/cureus.110305

**Published:** 2026-06-05

**Authors:** Sierra R Parkinson, Sabah Osmani, Claire Petitt, Paul B Googe, Rachel C Blasiak

**Affiliations:** 1 Department of Dermatology, University of North Carolina at Chapel Hill School of Medicine, Chapel Hill, USA

**Keywords:** arteriovenous hemangiomas, chronic liver disease, cutaneous vascular lesions, hepatic dysfunction, liver transplantation

## Abstract

Dermatologic abnormalities are common in patients with hepatic disorders, with the skin being one of the major target organs for extrahepatic manifestations. Common skin findings in liver disease include spider angiomas, palmar erythema, caput medusa, and nail changes. This report details a case of arteriovenous hemangiomas in a patient with alcohol-induced chronic liver disease. The patient received an orthotopic liver transplant, and follow-up 10 weeks later showed a marked reduction in the size of the arteriovenous hemangiomas as well as significant improvement of spider angiomas. Abnormal skin findings can be the first patient-reported symptoms or findings on exam. The marked reduction in this patient’s arteriovenous hemangiomas after liver transplantation supports prior evidence that treating underlying liver disease leads to arteriovenous hemangioma regression. While most cutaneous conditions are asymptomatic, familiarity and prompt recognition of common and uncommon skin changes of liver dysfunction, including arteriovenous hemangiomas, are important for early diagnosis and treatment initiation.

## Introduction

Arteriovenous hemangioma (AVH) is a rare acquired vascular tumor characterized histologically by a proliferation of thick- and thin-walled blood vessels, lined by a single layer of endothelial cells in the upper and mid-reticular dermis. Although the pathogenesis of AVH is not fully understood, leading hypotheses include a possible hamartomatous proliferation of the suprapapillary vascular plexus or a hamartoma of the Sucquet-Hoyer canal of the glomus body [1,2]. Clinically, AVHs typically present as an asymptomatic, solitary 0.5-1.0 cm well-circumscribed bluish-red papule or nodule on the head, neck, trunk, or extremities [1,2].

Chronic liver disease is associated with a broad spectrum of cutaneous manifestations reflecting systemic hepatic dysfunction, including jaundice, pruritus, and xanthelasma. Spider angiomas are the most representative and classic vascular lesion of chronic liver disease, with a reported prevalence of 33%, followed by palmar erythema at 23% [3]. AVHs remain exceedingly rare and underrecognized as part of the dermatologic spectrum of hepatic dysfunction; thus, the true prevalence of AVHs in patients with chronic liver dysfunction is unknown [1,2,4].

The exact mechanism linking AVH and chronic liver dysfunction is also poorly understood. A small number of cases exist in the literature describing AVHs in chronic liver disease, hypothesizing the potential roles of hormonal dysregulation of estrogen resulting from impaired hepatic metabolism, angiogenic dysregulation, and vascular remodeling associated with portal hypertension [1,2].

We present a case of multiple AVHs in a patient with alcohol-associated cirrhosis that demonstrated marked regression following liver transplantation. This report highlights AVHs as a potentially reversible vascular manifestation of cirrhosis and further expands the spectrum of cutaneous findings associated with chronic liver disease.

This case report was previously presented as a poster at the 47th Annual Southeastern Consortium for Dermatology Meeting on October 5, 2024.

## Case presentation

**Figure 1:**
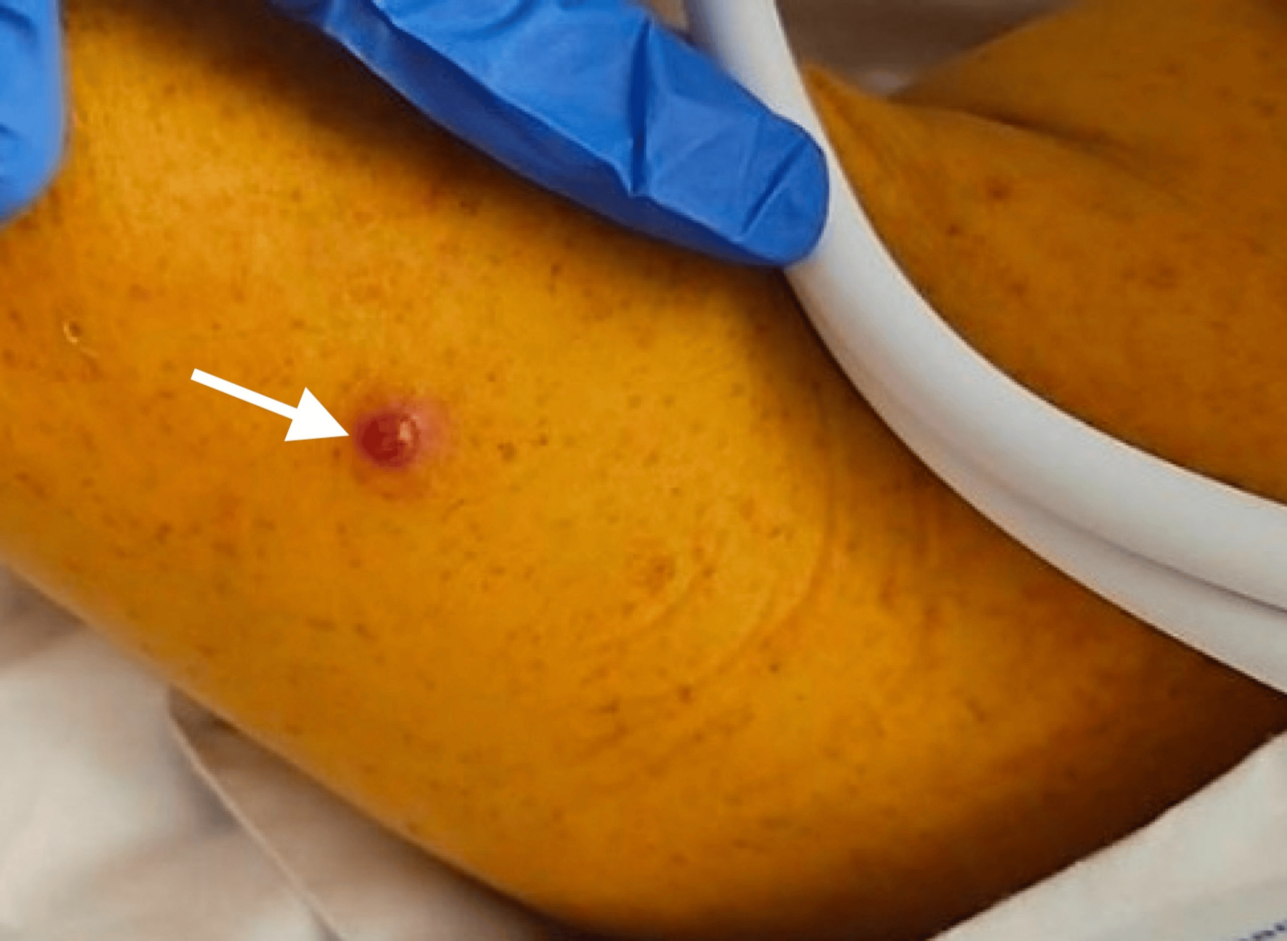
Initial presentation of a friable red vascular papule (white arrow) on the left forearm

**Figure 2:**
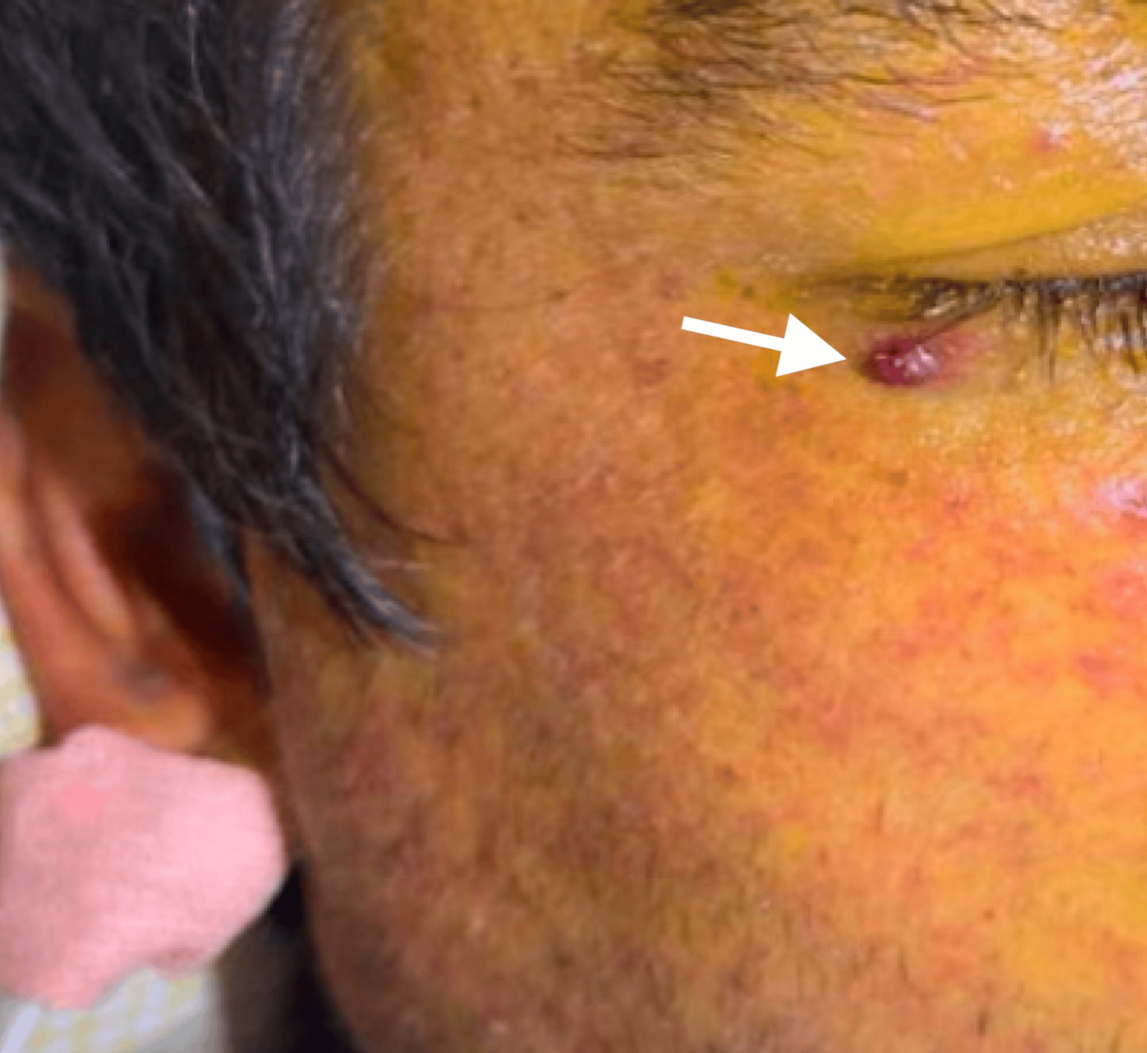
Initial presentation of a friable red vascular papule (white arrow) on the right lower eyelid

A 35-year-old male with a past medical history of alcoholic hepatitis and alcohol-induced cirrhosis was admitted to the hospital for acute liver failure in the setting of alcohol withdrawal. Dermatology was consulted to evaluate a worsening asymptomatic rash on his chest and two vascular papules on his forearm and lower eyelid that had been present for two months. Physical examination revealed three morphologically similar, well-circumscribed, 1 cm friable red vascular papules on the left forearm, right lower eyelid (Figures 1-2), and left chest, along with diffuse blanching vascular macules on the trunk. A separate red papule with a central eschar was present on the right upper arm.

**Figure 3:**
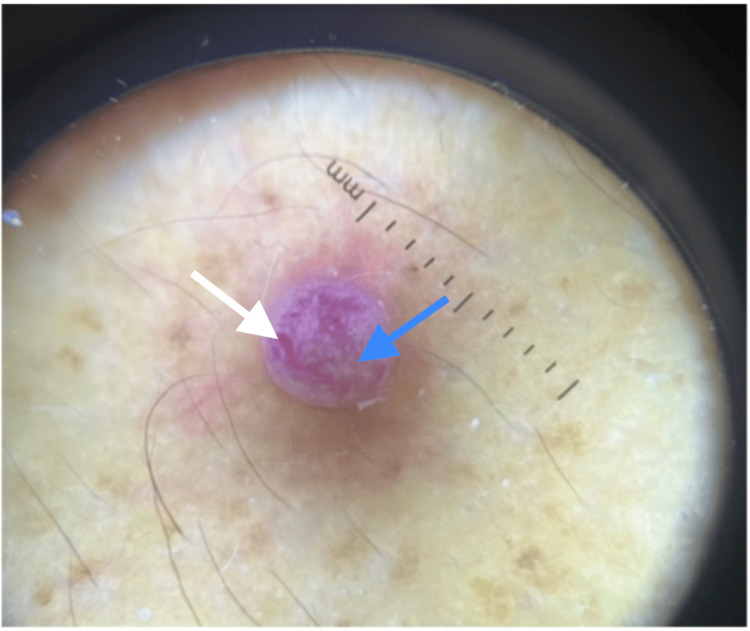
Dermascopic view of red friable vascular papule on the left forearm shows a round, well-defined papule with bright red, loosely organized lacunae (white arrow) set within a deep purplish background and traversed by white streaks (blue arrow)

Dermatoscopic evaluation of the left forearm lesion demonstrated a round, well-defined papule with bright red, loosely organized lacunae set within a deep purplish background and traversed by white streaks (Figure 3).

**Figure 4:**
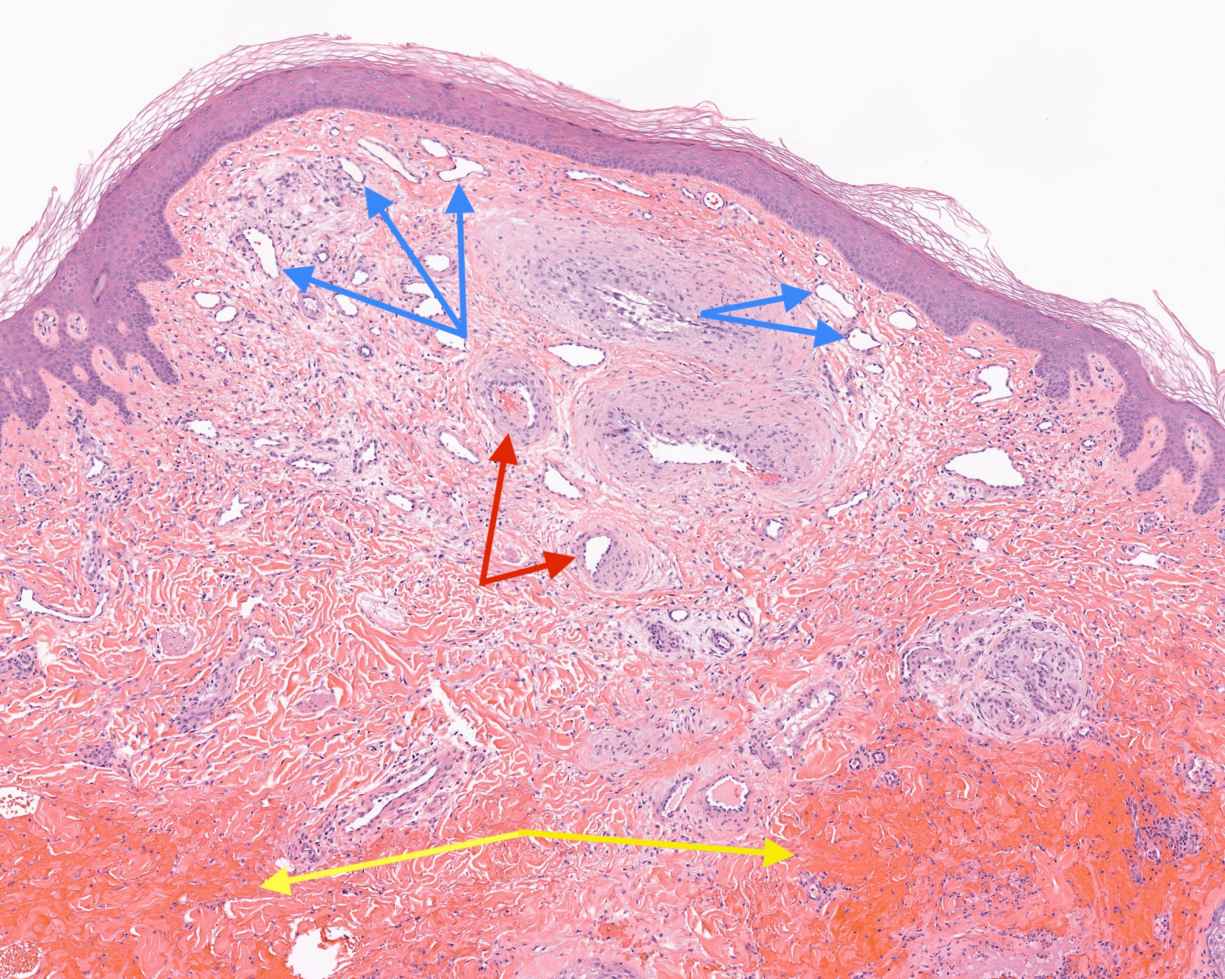
Histopathological findings (H&E stain, 10× magnification) of red friable vascular papule on left forearm demonstrating increased dermal vasculature composed of thick- (red arrows) and thin-walled (blue arrows) vessels with a prominent background of hemorrhage (yellow arrows) H&E: Hematoxylin and Eosin

Given the similar clinical appearance of the three vascular papules, the left chest lesion was selected for biopsy. Histopathologic examination demonstrated a papular architecture with increased dermal vasculature composed of thick- and thin-walled vessels with a prominent background of hemorrhage (Figure 4), consistent with an AVH. Based on the shared morphologic features of all three vascular papules, all were diagnosed as AVHs.

The blanching vascular macules were clinically consistent with spider angiomas. Fungal and acid-fast bacilli tissue cultures of the right arm lesion were obtained and were negative.

**Figure 5:**
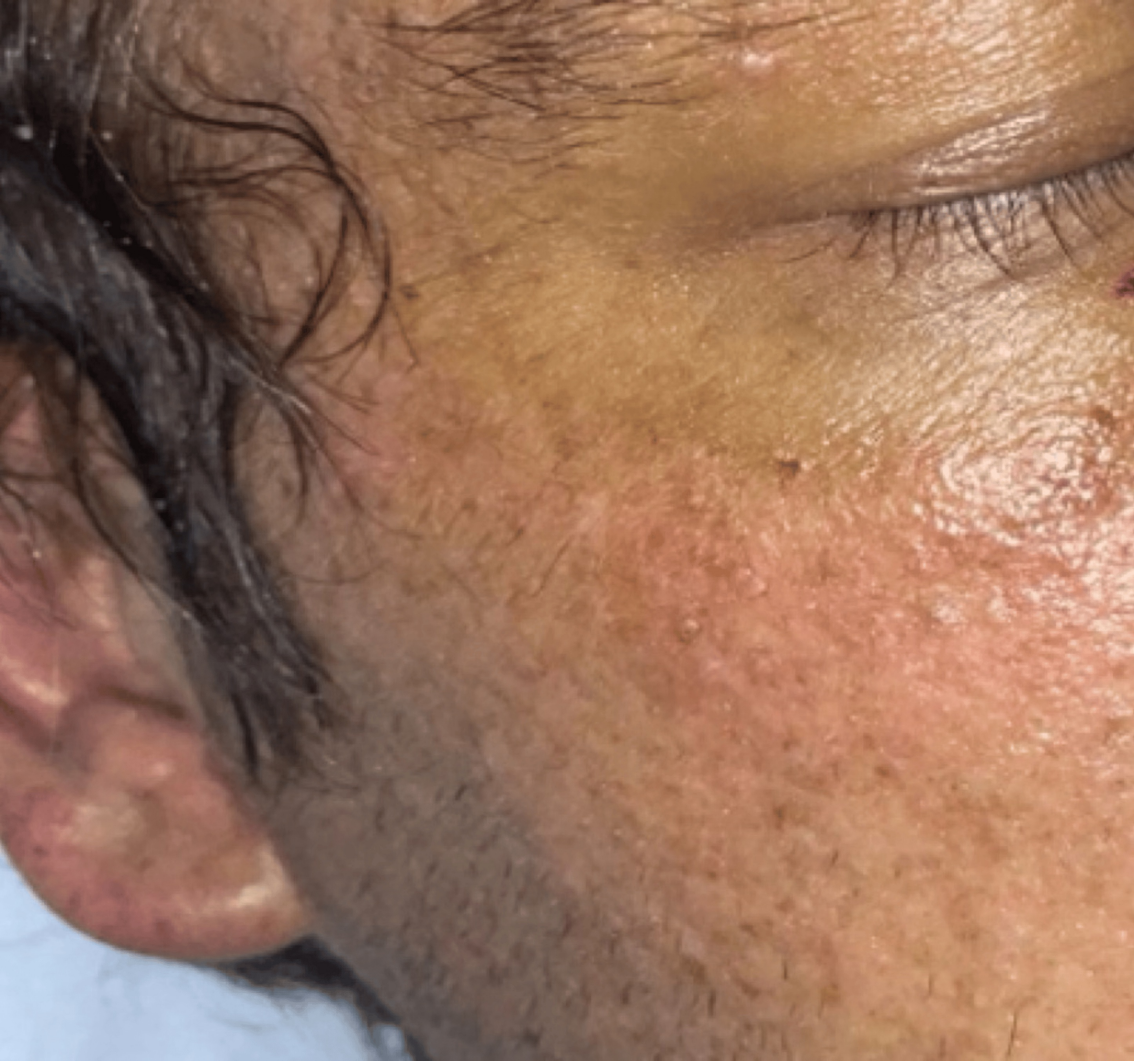
Follow-up image 10-weeks post initial liver transplant demonstrating complete resolution of right lower eyelid AVH AVH: Arteriovenous Hemangioma

**Figure 6:**
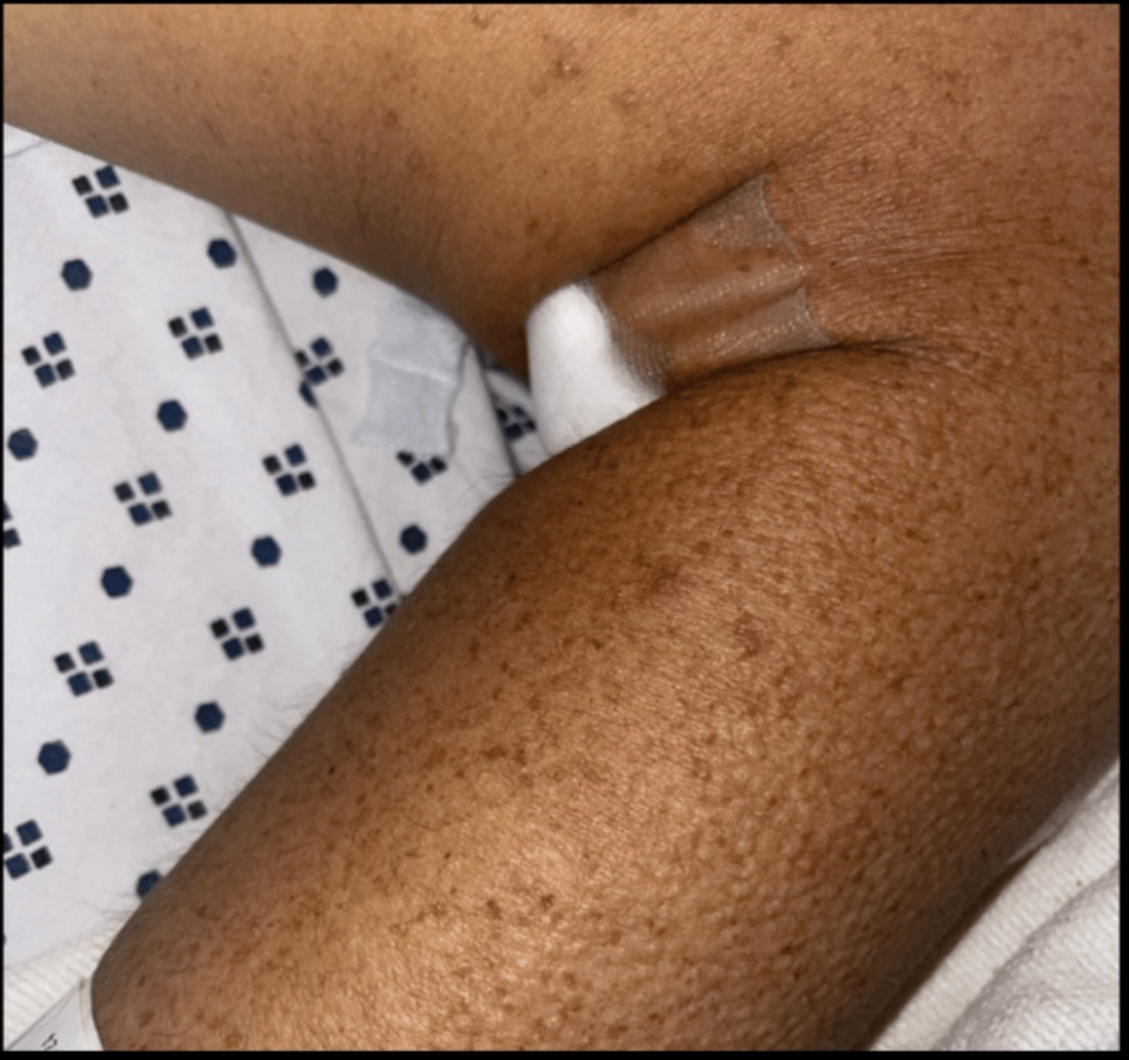
Follow-up image 10-weeks post initial liver transplant demonstrating complete resolution of left forearm AVH AVH: Arteriovenous Hemangioma

The patient received an orthotopic liver transplant complicated by rejection and bacteremia, followed by a simultaneous liver-kidney transplant nine weeks later. In a follow-up 10 weeks after the initial liver transplant, the patient remained asymptomatic, and the AVHs on the left forearm, right lower eyelid (Figures 5-6), and left chest had resolved completely and were no longer appreciable on gross examination. The diffuse spider angiomas likewise demonstrated complete clinical regression and were no longer visible. Given the absence of clinically detectable lesions, repeat dermatoscopy and histopathologic confirmation were not pursued. The dermatology team signed off, and the patient was subsequently lost to dermatologic follow-up after discharge.

## Discussion

Dermatologic abnormalities are common in patients with hepatic disorders, with the skin serving as a major target organ for extrahepatic manifestations of liver disease [3]. Prompt recognition of liver-related skin changes may aid in the identification of underlying hepatic pathology and facilitate earlier intervention. While spider angiomas, palmar erythema, and other vascular changes are well-established manifestations of cirrhosis [3], AVHs are less frequently recognized.

AVHs are benign acquired vascular tumors that typically present as bluish-red papules or nodules involving the head, trunk, and extremities and are reported most commonly in middle-aged men [1,2]. Given their rarity, the true prevalence of AVHs in cirrhotic patients is unknown.

Although an association between AVHs and chronic liver disease has been described, the literature is limited to isolated case reports and small case series [1,2,4], and the pathophysiologic mechanism remains incompletely understood. One proposed pathway involves hormonal dysregulation resulting from impaired hepatic metabolism. Elevated circulating estrogen levels have long been implicated in the development of vascular manifestations of cirrhosis, including spider angiomas and palmar erythema [1,3]. Given the clinical similarities between these lesions and AVHs, Lee et al. proposed that hyperestrogenism may contribute to AVH formation; however, immunohistochemical analysis failed to demonstrate estrogen receptor expression within lesional tissue, leaving the role of hormonal signaling uncertain [1].

Angiogenic dysregulation represents another plausible mechanism. Li et al. found elevated levels of vascular endothelial growth factor (VEGF) and basic fibroblast growth factor (bFGF) in patients with cirrhosis and spider angiomas, suggesting that chronic liver disease may promote a proangiogenic environment favorable to vascular proliferation [5]. Although these mediators have not been directly studied in AVHs, persistent angiogenic signaling may contribute to abnormal vascular growth and remodeling. Chronic hepatic dysfunction may also impair clearance of proangiogenic factors and promote endothelial activation, further facilitating vascular lesion development. Additionally, portal hypertension and chronic venous congestion may contribute to vascular remodeling through altered hemodynamic forces, resulting in proliferation of vessels of venous origin in cirrhosis-associated AVHs [2]. Together, these observations suggest that hormonal dysregulation, angiogenic signaling, and vascular remodeling may act synergistically in the development of AVHs. Akiyama and Inamoto’s observation of cirrhosis-associated AVHs in patients with alcohol-related liver disease and chronic hepatitis B or C infection supports this hypothesis, suggesting AVHs may be related to chronic hepatic dysfunction itself rather than a specific underlying liver disease etiology [2].

The marked regression of AVHs observed following liver transplantation in our patient supports this dynamic relationship between hepatic dysfunction and AVH development. Improvement occurred in parallel with regression of numerous spider angiomas, raising the possibility that these lesions share common pathogenic pathways. Restoration of hepatic function may normalize circulating hormonal and angiogenic mediators, reduce portal hypertensive vascular stress, and reverse the biologic stimuli responsible for maintaining these vascular proliferations. Thus, AVHs may represent a reversible manifestation of the vascular phenotype of chronic liver disease rather than a purely static benign neoplasm.

This case expands the limited literature describing AVHs in chronic liver disease and highlights the importance of recognizing AVHs as a potential cutaneous marker of hepatic dysfunction. Increased awareness among clinicians can facilitate earlier recognition of underlying liver disease, particularly when AVHs occur alongside more established stigmata such as spider angiomas. Further studies evaluating angiogenic mediators, such as VEGF and bFGF, along with hormonal pathways and hemodynamic factors in patients with AVHs may help clarify their pathogenesis and determine whether these lesions have utility as biomarkers of liver disease activity or treatment response.

## Conclusions

AVH is a rare and likely underrecognized dermatologic manifestation of chronic liver disease. The marked regression of all AVHs and spider angiomas following liver transplantation supports a possible pathophysiologic link between hepatic dysfunction and these cutaneous vascular lesions. The observations in this report align with prior hypotheses correlating hormonal, angiogenic, and hemodynamic derangements in cirrhosis to AVH development. Familiarity with this association and recognition of AVHs, especially alongside other cutaneous or systemic stigmata of liver disease, is vital for specialties and general practitioners as it may provide an important clue to underlying hepatic dysfunction, prompting timely evaluation and intervention. Future studies investigating the molecular mechanisms underlying AVH formation, such as VEGF and bFGF, in chronic liver dysfunction are needed to determine their potential role as biomarkers of disease activity and treatment response.
